# The stability and change of etiological influences on depression, anxiety symptoms and their co-occurrence across adolescence and young adulthood

**DOI:** 10.1017/S0033291715001634

**Published:** 2015-08-27

**Authors:** M. A. Waszczuk, H. M. S. Zavos, A. M. Gregory, T. C. Eley

**Affiliations:** 1 King's College London, MRC Social, Genetic and Developmental Psychiatry Centre, Institute of Psychiatry, Psychology and Neuroscience, London, UK; 2 Department of Psychology, Goldsmiths, University of London, London, UK

**Keywords:** Adolescence, anxiety, depression, development, generalized anxiety, genetics, panic, separation anxiety, social anxiety, twins, young adulthood

## Abstract

**Background.:**

Depression and anxiety persist within and across diagnostic boundaries. The manner in which common *v.* disorder-specific genetic and environmental influences operate across development to maintain internalizing disorders and their co-morbidity is unclear. This paper investigates the stability and change of etiological influences on depression, panic, generalized, separation and social anxiety symptoms, and their co-occurrence, across adolescence and young adulthood.

**Method.:**

A total of 2619 twins/siblings prospectively reported symptoms of depression and anxiety at mean ages 15, 17 and 20 years.

**Results.:**

Each symptom scale showed a similar pattern of moderate continuity across development, largely underpinned by genetic stability. New genetic influences contributing to change in the developmental course of the symptoms emerged at each time point. All symptom scales correlated moderately with one another over time. Genetic influences, both stable and time-specific, overlapped considerably between the scales. Non-shared environmental influences were largely time- and symptom-specific, but some contributed moderately to the stability of depression and anxiety symptom scales. These stable, longitudinal environmental influences were highly correlated between the symptoms.

**Conclusions.:**

The results highlight both stable and dynamic etiology of depression and anxiety symptom scales. They provide preliminary evidence that stable as well as newly emerging genes contribute to the co-morbidity between depression and anxiety across adolescence and young adulthood. Conversely, environmental influences are largely time-specific and contribute to change in symptoms over time. The results inform molecular genetics research and transdiagnostic treatment and prevention approaches.

## Introduction

Depression and anxiety disorders commonly co-occur (Angold *et al.* 1999; Costello *et al.* 2003; Kessler *et al.* 2005*b*; Beesdo *et al.* 2009; Gregory *et al.* 2007) and share multiple risk factors (Axelson & Birmaher, 2001), including substantial genetic overlap (Kendler *et al.* 1987; Thapar & McGuffin, 1997; Eley & Stevenson, 1999; Mosing *et al.* 2009; Zavos *et al.* 2012; Waszczuk *et al.* 2014). Both are chronic and show *homotypic* (within-disorder) and *heterotypic* (across-disorder) continuity over time (Merikangas, 1993; Pine *et al.* 2001; Costello *et al.* 2003; Goodwin *et al.* 2004; Rutter *et al.* 2006; Ferdinand *et al.* 2007; Gregory *et al.* 2007; Moffitt *et al.* 2007; Trzaskowski *et al.* 2011; Lahey *et al.* 2014). Homotypic continuity suggests a degree of specificity of the constructs, in line with DSM-5 categorization (APA, 2013), in that the construct shows a fairly stable presentation. Additionally, heterotypic continuity also indicates an overlap between them, as proposed by transdiagnostic theories (Insel *et al.* 2010; Wilamowska *et al.* 2010). Furthermore, there are developmental differences in the phenotypic and genetic relationship between depression and *different* anxiety disorders (Axelson & Birmaher, 2001; Bergen *et al.* 2007; Moffitt *et al.* 2007; Goldberg, 2008; Hettema, 2008; Mennin *et al.* 2008; Beesdo *et al.* 2009, 2010; Waszczuk *et al.* 2014). For example, we recently found age differences in phenotypic and genetic overlap between depression and a range of anxiety symptoms, with the association between these symptoms increasing markedly from adolescence, indicating developmentally dynamic etiology of internalizing problems (Waszczuk *et al.* 2014). As a result, genetic and environmental influences are likely to vary in their contribution to the continuity of depression, different anxiety subtypes, and their longitudinal co-occurrence across time. Understanding how these risk and maintenance factors operate across development is crucial for informing successful prevention and intervention strategies. Thus, the current study investigated the continuity and change of genetic and environmental influences on homotypic and heterotypic continuity of depression and four anxiety symptom scales across adolescence and young adulthood.

### Homotypic continuity

To date longitudinal twin studies have focused largely on the contribution of genetic and environmental influences to homotypic continuity of depression, anxiety, or composite internalizing symptoms. Some studies have found that stable genetic influences contribute substantially to homotypic continuity across the lifespan (O'Connor *et al.* 1998; Gillespie *et al.* 2004; Trzaskowski *et al.* 2011; Garcia *et al.* 2013; Waszczuk *et al.* 2013). Conversely, other studies, primarily those of children and adolescents, have found that alongside genetic stability, new genetic influences emerge that contribute to change in symptoms over time (Scourfield *et al.* 2003; Van Der Valk *et al.* 2003; Bartels *et al.* 2004; Haberstick *et al.* 2005; Lau & Eley, 2006; Kendler *et al.* 2008*a*, *b*; Zavos *et al.* 2012; Nivard *et al.* 2015; Lewis & Plomin, 2015). This is in line with evidence that childhood and adolescence are more genetically dynamic than adulthood (Nivard *et al.* 2015).

The role of shared environmental influences is also unclear. Evidence from child samples suggests that stable shared environmental influences contribute to the homotypic continuity of symptoms (Schmitz *et al.* 1995; Silberg *et al.* 2001; Scourfield *et al.* 2003; Van Der Valk *et al.* 2003; Bartels *et al.* 2004; Kendler *et al.* 2008*a*), as well as to heterotypic continuity between different anxiety traits across time (Trzaskowski *et al.* 2011). This has generally not been replicated in older twins, possibly because shared environmental influences play a diminishing role in adolescence and adulthood (Rapee *et al.* 2009). Finally, non-shared environmental influences tend to be time-specific and contribute to change rather than stability of internalizing symptoms over time (Scourfield *et al.* 2003; Van Der Valk *et al.* 2003; Bartels *et al.* 2004; Haberstick *et al.* 2005; Lau & Eley, 2006; Zavos *et al.* 2012; Garcia *et al.* 2013; Lewis & Plomin, 2015). However, some studies have found that non-shared environmental influences can contribute to the homotypic continuity of depression and anxiety symptoms (O'Connor *et al.* 1998; Kendler *et al.* 2008*a*, 2011; Nivard *et al.* 2015).

Despite remarkable heterogeneity of anxiety disorders, to our knowledge only two studies to date have investigated the etiology of homotypic continuity of different anxiety symptoms. The first study examined three types of phobia from childhood to adulthood, and found more stable shared environmental influences on animal than situational and blood/injury fears (Kendler *et al.* 2008*a*). The second study investigated genetic and environmental influences on panic, separation and generalized anxiety symptoms across middle childhood, and found genetic stability and largely time-specific environmental influences consistently in the three symptoms (Waszczuk *et al.* 2013). To address this gap in the literature, the first aim of the current study was to systematically explore and compare the genetic and environmental influences on the homotypic continuity of depression and four anxiety symptom scales – panic, generalized, separation and social anxiety. We focused specifically on adolescence and young adulthood, developmental periods characterized by increased prevalence of depression and some of the anxiety disorders (Costello *et al.* 2003), and a time of substantial maturation of emotional processing (Yurgelun-Todd, 2007; Blakemore, 2008; Kadosh *et al.* 2013). In line with certain previous studies in adolescents we hypothesized that: (i) stable genetic factors would substantially contribute to homotypic continuity of each symptom in this age group, (ii) there would be time-specific genetic and environmental influences that contribute to change in the course of each symptom. We also explored whether there would be differences in the etiology of homotypic continuity across time between the symptom scales.

### Heterotypic continuity

To date only two studies have examined how dynamic changes in etiological influences contribute to the co-morbidity of internalizing disorders over time. The first study found that common genetic influences on childhood overanxious disorder and phobias continue to adolescence, where they also predict variance in adolescent depression (Silberg *et al.* 2001). Furthermore, shared environmental influences contributed to heterotypic continuity between some of the internalizing symptoms. The second study found that the genetic influences on childhood separation anxiety disorder continue to influence adult onset panic attacks (Roberson-Nay *et al.* 2012). However, the degree to which stable and time-specific etiological influences are shared between depression and anxiety disorder symptoms across development remains largely unknown. Understanding how genetic and environmental influences contribute to the co-morbidity of internalizing symptoms over time might provide clinically-relevant insights in the context of growing interest in transdiagnostic interventions. Given a remarkably high genetic overlap and small to moderate non-shared environmental correlations between these multiple disorders (Kendler *et al.* 1987; Eley & Stevenson, 1999; Thapar & McGuffin, 1997; Spatola *et al.* 2007; Mosing *et al.* 2009; Zavos *et al.* 2012; Waszczuk *et al.* 2014), we tentatively hypothesized that: (iii) both stable and time-specific genetic influences would contribute to the longitudinal co-morbidity between depression and anxiety symptom scales, (iv) environmental influences would not contribute markedly to the longitudinal co-morbidity.

## Method

### Participants

The analyses use data from waves 2–4 (hereafter referred to as times 1–3 respectively) of a longitudinal twin and sibling study, the Genesis 1219 (G1219). Full details are provided elsewhere (McAdams *et al.* 2013) (see Supplementary Method). The study was given ethical approval by the Research Ethics Committee of the Institute of Psychiatry, King's College, London, South London and Maudsley NHS Trust and Goldsmiths, University of London. Informed consent was obtained from parents of adolescents under 16 years and from participants over 16. The sample size at time 1 was 1372 pairs [350 monozygotic (MZ), 313 dizygotic same-sex (DZss), 334 dizygotic opposite-sex (DZos), 330 siblings; 56% female; mean age 15 years (range 12–21, s.d. = 1.67)], at time 2 it was 866 pairs [234 MZ, 207 DZss, 232 DZos, 182 siblings; 60% female; mean age 17 years (range 14–23, s.d. = 1.67)], and at time 3 it was 896 pairs [230 MZ, 214 DZss, 232 DZos, 201 siblings; 61% female; mean age 20 years (range 18–27, s.d. = 1.76)]. The inclusion of siblings inevitably resulted in large age ranges; however, 72% of the participants were twins with a tighter age range (e.g. at time 2, age s.d. = 1.11, range = 15–19 for twins, age s.d. = 1.97, range = 15–23 for siblings). Attrition was predicted by socioeconomic status (responses were more likely from individuals with parents reporting higher qualifications and home ownership), delinquency (individuals reporting lower levels of delinquent behavior were more likely to stay in the study) and sex (females were more likely than males to remain in the study), but not by zygosity and internalizing symptoms.

### Measures

#### Depression symptoms

At each time the participants completed the Short Mood and Feelings Questionnaire (Angold *et al.* 1995), a 13-item self-report measure assessing how often depression symptoms occurred in the past 2 weeks. Responses were summed to give total depression scores. The measure demonstrates good reliability and validity (Angold *et al.* 1995) and the internal consistency was very high in the current study (*α* = 0.79–0.90).

#### Anxiety symptoms

The adolescents (times 1 and 2) completed the Spence Children's Anxiety Scale (Spence, 1998); a 38-item self-report questionnaire tapping common anxiety symptoms. Adults (time 3) completed the Revised Symptoms of Anxiety Scale (see Gregory *et al.* 2011), an age-appropriate version of the Revised Child Anxiety and Depression Scale (Chorpita *et al.* 2000), consisting of 36 self-report items designed to assess DSM-IV anxiety disorder symptoms. Responses were summed to create four DSM-IV-related anxiety symptom scales: panic, generalized, separation and social anxiety. Subscales were originally derived using exploratory factor analyses conducted in large, independent samples (Spence, 1997, 1998; Chorpita *et al.* 2000). All measures have good internal consistency (*α* = 0.66–0.77 for separation anxiety, *α* = 0.70–0.90 for all other scales) (Spence, 1998; Birmaher *et al.* 1999; Chorpita *et al.* 2000; Gregory *et al.* 2011).

The internal consistencies and descriptive statistics of all measures were comparable to published samples and are presented elsewhere (Waszczuk *et al.* 2014).

### Analyses

The twin design compares the similarity between MZ (sharing 100% of their genes) and DZ (sharing on average 50% of their segregating genes) twin pairs. Differences in within-pair correlations allows estimations of the influences of additive genetics (A), shared environment (C) factors that contribute to phenotypic similarity between siblings) and non-shared environment (E, factors that contribute to phenotypic differences between siblings). Quantitative genetic methods are described comprehensively elsewhere (Rijsdijk & Sham, 2002; Plomin *et al.* 2013).

Models were fitted using OpenMx (Boker *et al.* 2011) within R [http://www.R-project.org (Team RDC, 2010)], a structural equation modeling package for genetically informative data. As is standard in model fitting analysis, variables were regressed for age and sex (McGue & Bouchard, 1984), and any with skew >1 were transformed.

Models were fitted using raw data maximum likelihood. The core fit statistic was minus twice the log likelihood (–2LL) of the observations. This is not an overall measure of fit, but provides a relative measure of fit, since differences in –2LL between models are distributed as *χ*^2^. To examine the overall fit of the genetic model we compared the –2LL to that of a saturated model (which fully describes data using the maximum number of free parameters, estimating variances, covariances and means for the raw data to get a baseline index of fit). The fit of sub-models was assessed by *χ*^2^ difference tests, i.e. Akaike's Information Criterion (AIC) and Bayesian Information Criterion, with lower values suggesting a better fit. If the difference between AIC of two models was <10, the more parsimonious model was selected (Wagenmakers & Farrell, 2004). Information about the precision of parameter estimates was obtained by likelihood-based confidence intervals.

Univariate genetic analyses were conducted on all variables at each time. Males and females showed differences in variance on all variables except for social anxiety, and a scalar was fitted to account for this difference (Waszczuk *et al.* 2014). Quantitative sex differences were tested to see whether males and females differ in magnitude of genetic and environmental influences, but such differences were not found. Finally, comparisons indicated that covariances, means and variances could be equated across DZ twins and siblings for all variables.

### Homotypic continuity

**Fig. 1.**
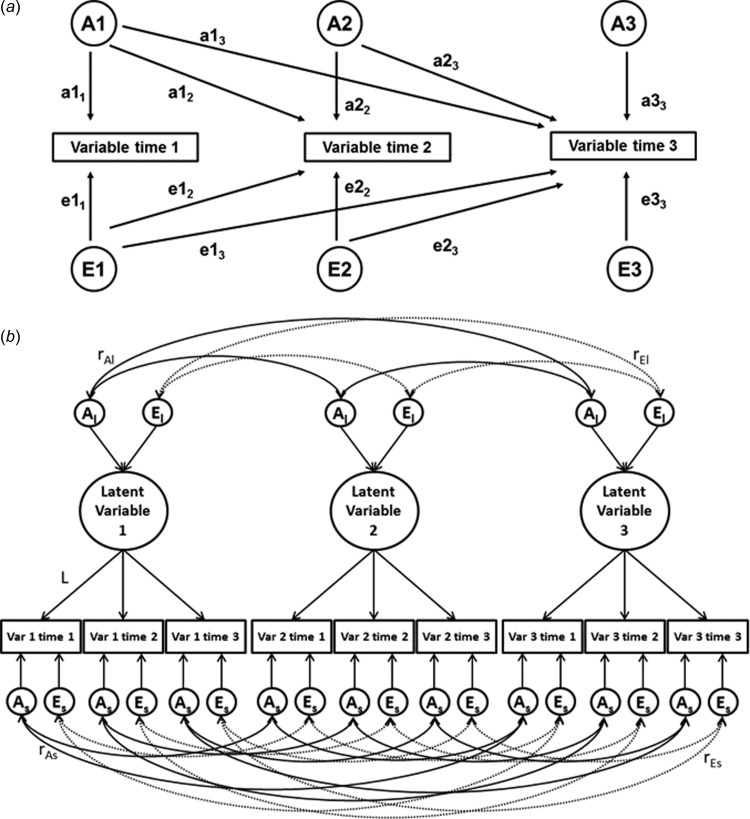
Multivariate models: (*a*) longitudinal Cholesky decomposition, (*b*) Common pathway model. A, Additive genetic effects; E, non-shared environmental effects, Var, variable. Subscript ‘l’ denotes stable influences on the latent factor, subscript ‘s’ denotes variable- and time-specific influences. In panel (*a*), variance paths, which must be squared to estimate the proportion of variance accounted for, are represented by lowercase letters and followed by two numerals, e.g. a1_1_, c2_2_, e3_3_. In panel (*b*), only three variables are presented for clarity; however the model was run with all five variables included, each measured at three time points.

Multivariate models best suited to investigate specific research questions were chosen a priori. The Cholesky decomposition (Fig. 1*a*) was used to examine the *homotypic continuity* of etiological influences separately for each variable. The Cholesky decomposition assumes three distinct sets of genetic and environmental influences on a variable at each time point. A1 and E1 are common factors on the first variable (paths a1_1_ and e1_1_) that can also influence the remaining two variables (paths a1_2–3_ and e1_2–3_, reflecting continuity from time 1 to times 2 and 3). A2 and E2 influence the second variable (paths a2_2_ and e2_2_, reflecting new genes emerging at time 2) and can also influence the third variable over and above the influences accounted for by A1 and E1 (paths a2_3_ and e2_3_, reflecting continuity from time 2 to time 3). A3 and E3 are unique influences specific to the third variable only (paths a3_3_ and e3_3_, reflecting new influences emerging at time 3). Total A and E effects on each individual measure can be obtained by summing all squared paths to that measure (e.g. the proportion of total variance in third variable explained by A influences is obtained by summing squared paths a1_3_, a2_3_ and a3_3_).

### Heterotypic continuity

The common pathway model (Fig. 1*b*) was fitted in order to investigate the stability and change of the etiological influences shared between depression and anxiety symptom scales across development, to inform the mechanisms underpinning *heterotypic continuity* across time. The model is illustrated on Fig. 1*b* (with just three variables for clarity); the model was run with all five variables included, each measured at three time points. This model assumes five latent factors; each underlying a variable assessed three times. For example, the depression latent factor captures the stability of the depression symptoms across times 1–3. Variance of each latent factor is then decomposed into genetic (A_l_) and environmental (E_l_) influences to assess the etiological factors underpinning the stability of each symptom. Of note, E_l_ is free from time-specific measurement error but not from shared measurement error. The genetic and environmental correlations between the latent factors (*r*_Al_ and *r*_El_) represent the degree of developmental stability common to depression and anxiety symptom scales. Any remaining variance (not explained by the latent factor) is then calculated as variable-specific genetic and environmental influences (A_s_ and E_s_). The variable-specific etiological influences include genetic and environmental influences that emerge at later time points, and are allowed to correlate with the within-time influences on all other variables (*r*_As_ and *r*_Es_), capturing time-specific associations between them.

## Results

### Phenotypic correlations

The longitudinal correlations between the variables across the three time points are presented in Table 1. All variables showed moderate homotypic continuity (*r* = 0.35–0.58). The heterotypic correlations between the different anxiety symptom scales, and between depression and each of the anxiety scales, were similar in magnitude and generally moderate (*r* = 0.12–0.46 and *r* = 0.11–0.39, respectively). Homotypic correlations were generally larger than heterotypic correlations, but tended to decrease at longer time intervals (times 1–3).

**Table 1.** Longitudinal phenotypic correlations

	Depression	Panic	Generalized anxiety	Separation anxiety	Social anxiety
Time 2 (17 years)	Time 1 (15 years)				
Depression	**0.47** (**0.43–0.51)**	0.33 (0.29–0.37)	0.31 (0.27–0.35)	0.24 (0.19–0.29)	0.30 (0.26–0.34)
Panic	0.31 (0.27–0.35)	**0.43 (0.39–0.47)**	0.32 (0.28–0.36)	0.24 (0.19–0.29)	0.21 (0.16–0.26)
Generalized anxiety	0.37 (0.33–0.41)	0.39 (0.35–0.43)	**0.47 (0.43–0.51)**	0.37 (0.33–0.41)	0.35 (0.31–0.39)
Separation anxiety	0.11 (0.06–0.16)	0.22 (0.17–0.27)	0.22 (0.17–0.27)	**0.36 (0.32–0.40)**	0.16 (0.11–0.21)
Social anxiety	0.29 (0.24–0.33)	0.29 (0.24–0.33)	0.34 (0.30–0.38)	0.31 (0.27–0.35)	**0.53 (0.49–0.56)**
Time 3 (20 years)	Time 1 (15 years)				
Depression	**0.38 (0.34–0.42)**	0.32 (0.28–0.36)	0.26 (0.21–0.31)	0.24 (0.19–0.29)	0.24 (0.19–0.29)
Panic	0.30 (0.26–0.34)	**0.39 (0.35–0.43)**	0.36 (0.32–0.40)	0.28 (0.23–0.33)	0.26 (0.21–0.31)
Generalized anxiety	0.34 (0.30–0.38)	0.33 (0.29–0.37)	**0.36 (0.32–0.40)**	0.35 (0.31–0.39)	0.34 (0.30–0.38)
Separation anxiety	0.32 (0.28–0.36)	0.40 (0.36–0.44)	0.35 (0.31–0.39)	**0.39 (0.35–0.43)**	0.32 (0.28–0.36)
Social anxiety	0.33 (0.29–0.37)	0.33 (0.29–0.37)	0.38 (0.34–0.42)	0.31 (0.27–0.35)	**0.46 (0.42–0.50)**
Time 3 (20 years)	Time 2 (17 years)				
Depression	**0.47 (0.43–0.51)**	0.34 (0.30–0.38)	0.38 (0.34–0.42)	0.12 (0.07–0.18)	0.31 (0.27–0.35)
Panic	0.34 (0.30–0.38)	**0.48 (0.44–0.52)**	0.45 (0.41–0.49)	0.21 (0.16–0.26)	0.28 (0.23–0.33)
Generalized anxiety	0.39 (0.35–0.43)	0.31 (0.27–0.35)	**0.53 (0.49–0.56)**	0.25 (0.20–0.30)	0.40 (0.36–0.44)
Separation anxiety	0.35 (0.31–0.39)	0.40 (0.36–0.44)	0.46 (0.42–0.50)	**0.35 (0.31–0.39)**	0.36 (0.32–0.40)
Social anxiety	0.39 (0.35–0.43)	0.30 (0.26–0.34)	0.46 (0.42–0.50)	0.17 (0.12–0.22)	**0.58 (0.54–0.62)**

Mean ages provided in the headings.

Homotypic continuity is presented on diagonal (in bold), heterotypic continuity across diagonal.

95% confidence intervals (CIs) are presented in parentheses. CIs not inclusive of zeros indicate significant correlations. Non-overlapping CIs mean significant difference between the values.

Results presented on untransformed variables for comparison with other published samples. The within-time correlations between depression and anxiety subscales are discussed elsewhere (Lau *et al.* 2007; Waszczuk *et al.* 2014). The homotypic continuity of anxiety subscales has also previously been reported (Waszczuk *et al.* 2014).

### Homotypic continuity

**Fig. 2.**
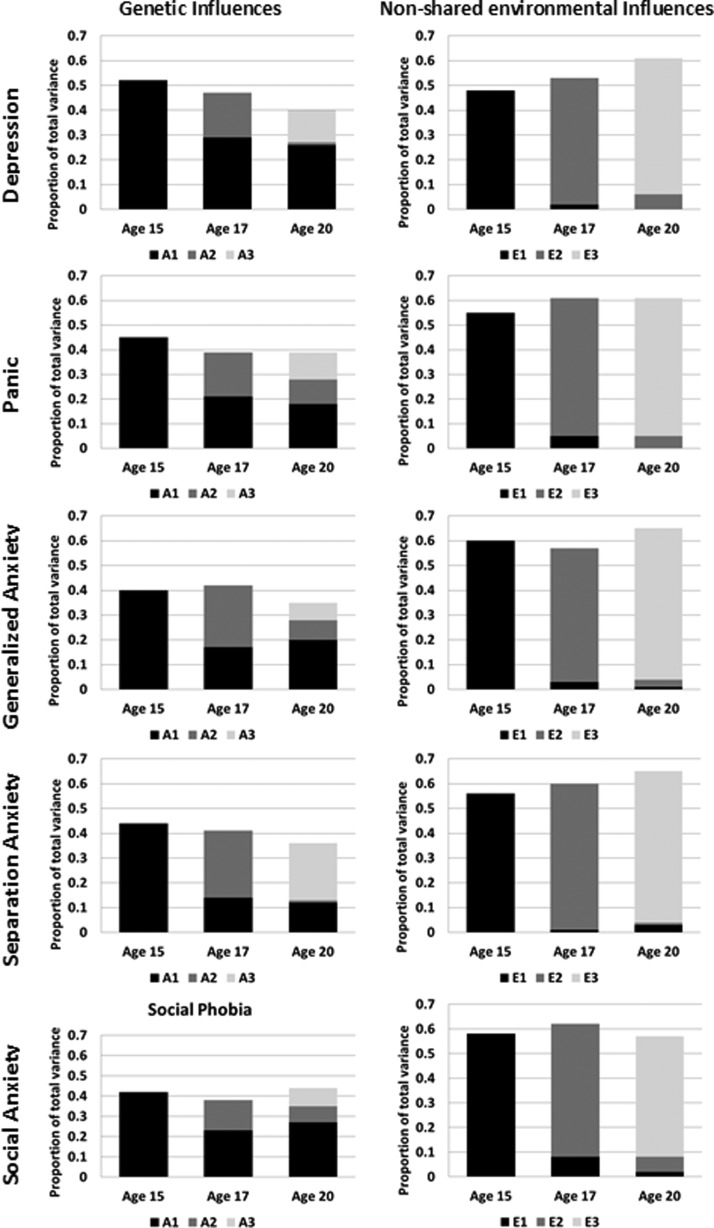
Longitudinal Cholesky decomposition results: The proportion of total variance in depression and anxiety symptom scales accounted for by genetic and non-shared environmental influences. A, Additive genetic effects; E, non-shared environmental influences. Mean ages provided in the x-axis. The y-axis represents the total phenotypic variance so the sum of all the factors equals the total heritability/non-shared environmental influences. The first genetic/non-shared environmental factor (A1/E1), which influences a variable at mean age 15, is represented in black. A dark gray represents the second genetic/non-shared environmental factor (A2/E2) that stars at mean age 17 years and the pale gray represents the third genetic/non-shared environmental factor (A3/E3) that emerges at mean age 20 years. The 95% confidence intervals are presented in Supplementary Table S1. AE models are presented, as C influences were not significant and were dropped from the multivariate models without a significant deterioration of the fit (Supplementary Table S2). The AIC values suggest that dropping C leads to improvement of the model fit at these three waves. Full ACE results are presented in Supplementary Tables S3–S5 for completeness.

The Cholesky decompositions show the effect of stable and new genetic and environmental factors across the three times, separately for each of the five symptom scales. The results were similar for depression and each anxiety symptom scale (Fig. 2). First, there was evidence of substantial genetic continuity, whereby genetic factors influencing symptoms at any one age continue to affect the symptom at subsequent ages. Second, the early influences gradually declined over time. For example, the first set of genetic factors (corresponding to path a1_1_ on Fig. 1*a*) accounted for 45% of the variance in generalized anxiety symptoms at age 15, but reduced to 21% by age 17 (path a1_2_) and 18% by age 20 (path a1_3_). Third, new genetic factors emerged at each age (paths a2_2_ and a3_3_). Genetic influences that emerged at age 17 continued to influence symptoms at age 20 (path a2_3_) in generalized anxiety, panic and social anxiety, but not in depression and separation anxiety. Separation anxiety was characterized by particularly high change in genetic influences over time.

Non-shared environmental influences on all symptoms were largely age-specific. For example, the non-shared environmental factors influencing generalized anxiety symptoms at age 15 had a small effect at age 17, and no significant effect at age 20. For 95% confidence intervals see Supplementary Table S1.

### Heterotypic continuity

In the common pathway model the total variance in each variable is explained by the latent factor and the variable-specific influences. Stable influences accounted for 21–69% of the variance in each variable (L^2^, see Table 2 note) and were largely influenced by genes (A_l_ = 0.61–0.76), with the remaining variance explained by modest to moderate, significant non-shared environmental influences (E_l_ = 0.24–0.39) (Table 2). The latent factors were generally highly correlated (*r*_phl_ = 0.58–0.83) (Table 3). Genetic influences on latent factors overlapped considerably (*r*_Al_ = 0.60–0.86) and the non-shared environmental correlations between the latent factors were also high (*r*_El_ = 0.46–0.76) (Table 3). The variable-specific genetic influences were small (A_s_ = 0.01–0.26) (Table 2), since most of the genetic influences acted via the latent factors. Conversely, variable-specific non-shared environmental influences were moderate (E_s_ = 0.31–0.56) and accounted for most of the non-shared environmental influences on each variable (Table 2). The phenotypic within-time correlations between the variable-specific influences varied widely (*r*_phs_ = −0.12–0.56), as did the genetic and non-shared environmental within-time correlations between them (Table 3).

**Table 2.** Common pathway model results: genetic and non-shared environmental influences on the latent factor, and latent factor and time-specific influences on each variable

		Depression			Panic			Generalized anxiety			Separation anxiety			Social anxiety		
Etiological influences on the latent factor	A_l_	0.76 (0.65–0.85)			0.66 (0.43–0.77)			0.64 (0.51–0.74)			0.69 (0.53–0.83)			0.61 (0.51–0.71)		
	E_l_	0.24 (0.15–0.35)			0.34 (0.23–0.46)			0.36 (0.26–0.49)			0.31 (0.17–0.47)			0.39 (0.29–0.49)		
Mean age, years		15	17	20	15	17	20	15	17	20	15	17	20	15	17	20
Latent factor influences on each variable	L	0.63 (0.58–0.67)	0.78 (0.73–0.83)	0.58 (0.52–0.62)	0.56 (0.51–0.61)	0.71 (0.66–0.76)	0.61 (0.56–0.66)	0.57 (0.52–0.61)	0.83 (0.78–86)	0.58 (0.53–0.63)	0.55 (0.50–0.61)	0.46 (0.38–0.52)	0.60 (0.54–0.66)	0.61 (0.57–0.65)	0.82 (0.78–0.86)	0.68 (0.63–0.72)
Time-specific etiological influences on each variable	A_s_	0.20 (0.13–0.27)	0.03 (0.00–0.11)	0.14 (0.05–0.23)	0.17 (0.10–0.25)	0.12 (0.05–0.21)	0.07 (0.00–0.15)	0.22 (0.15–0.30)	0.01 (0.00–0.09)	0.12 (0.03–0.21)	0.21 (0.13–0.29)	0.26 (0.16–0.36)	0.11 (0.02–0.20)	0.17 (0.10–0.24)	0.02 (0.00–0.07)	0.11 (0.02–0.20)
	E_s_	0.41 (0.35–0.47)	0.36 (0.28–0.43)	0.53 (0.45–0.63)	0.51 (0.44–0.58)	0.37 (0.28–0.46)	0.56 (0.47–0.65)	0.45 (0.38–0.53)	0.31 (0.22–0.38)	0.54 (0.46–0.63)	0.48 (0.41–0.56)	0.53 (0.44–0.64)	0.53 (0.44–0.62)	0.45 (0.39–0.53)	0.31 (0.24–0.37)	0.44 (0.36–0.52)

A, Additive genetic effects; E, non-shared environmental effects; L, Latent factor.

Mean ages provided in the headings.

95% confidence intervals (CIs) are presented in parentheses. CIs not inclusive of zeros indicate significant influences. Non-overlapping CIs mean significant difference between the values.

L needs to be squared to inform about the proportion of total variance accounted for by the latent factor. L^2^ should be multiplied by A_l_ to obtain the proportion of the total variance due to the genetic influences from the latent factor. L^2^ should be multiplied by E_l_ to obtain the proportion of the total variance due to the non-shared environmental influences from the latent factor. Total variance of a trait = L^2^ + A_s_ + E_s_.

AE models are presented, as C influences were not significant and were dropped from the multivariate models without a significant deterioration of the fit (Supplementary Table S2). The AIC values suggest that dropping C lead to improvement of the model fit at these three waves. Full ACE results are presented in Supplementary Tables S3–S5 for completeness.

**Table 3.** Common pathway model results: phenotypic, genetic and non-shared environmental correlations between the latent factors and time-specific influences at 15, 17 and 20 years

	Depression	Panic	Generalized anxiety	Separation anxiety
Latent factors				
Panic				
*r*_phl_	0.72 (0.67 to 0.76)			
*r*_Al_	0.74 (0.66 to 0.83)			
*r*_El_	0.67 (0.46 to 0.85)			
Generalized anxiety				
*r*_phl_	0.74 (0.69 to 0.79)	0.83 (0.79 to 0.87)		
*r*_Al_	0.81 (0.73 to 0.88)	0.86 (0.79 to 0.94)		
*r*_El_	0.60 (0.41 to 0.76)	0.76 (0.61 to 0.89)		
Separation anxiety				
*r*_phl_	0.58 (0.51 to 0.65)	0.76 (0.70 to 0.82)	0.80 (0.75 to 0.85)	
*r*_Al_	0.60 (0.48 to 0.71)	0.78 (0.67 to 0.89)	0.86 (0.76 to 0.96)	
*r*_El_	0.55 (0.29 to 0.80)	0.73 (0.51 to 0.95)	0.70 (0.50 to 0.89)	
Social anxiety				
*r*_phl_	0.63 (0.58 to 0.68)	0.62 (0.56 to 0.67)	0.75 (0.71 to 0.79)	0.65 (0.59 to 0.70)
*r*_Al_	0.67 (0.59 to 0.76)	0.68 (0.57 to 0.78)	0.77 (0.69 to 0.85)	0.75 (0.64 to 0.87)
*r*_El_	0.56 (0.37 to 0.72)	0.52 (0.34 to 0.67)	0.71 (0.58 to 0.83)	0.46 (0.24 to 0.65)
Time-specific influences at 15				
Panic				
*r*_phs_	0.43 (0.38 to 0.48)			
*r*_As_	0.80 (0.59 to 0.99)			
*r*_Es_	0.28 (0.19 to 0.38)			
Generalized anxiety				
*r*_phs_	0.44 (0.39 to 0.48)	0.49 (0.44 to 0.52)		
*r*_As_	0.57 (0.37 to 0.74)	0.56 (0.32 to 0.73)		
*r*_Es_	0.38 (0.28 to 0.46)	0.46 (0.38 to 0.53)		
Separation anxiety				
*r*_phs_	0.34 (0.29 to 0.39)	0.40 (0.36 to 0.45)	0.43 (0.38 to 0.48)	
*r*_As_	0.40 (0.15 to 0.59)	0.64 (0.41 to 0.86)	0.58 (0.38 to 0.76)	
*r*_Es_	0.31 (0.22 to 0.41)	0.31 (0.22 to 0.40)	0.36 (0.27 to 0.44)	
Social anxiety				
*r*_phs_	0.36 (0.31 to 0.41)	0.37 (0.32 to 0.42)	0.48 (0.44 to 0.52)	0.46 (0.42 to 0.51)
*r*_As_	0.61 (0.38 to 0.81)	0.64 (0.39 to 0.88)	0.81 (0.62 to 0.99)	0.66 (0.44 to 0.86)
*r*_Es_	0.26 (0.16 to 0.36)	0.28 (0.18 to 0.37)	0.35 (0.25 to 0.43)	0.39 (0.29 to 0.47)
Time-specific influences at 17				
Panic				
*r*_phs_	0.19 (0.09 to 0.28)			
*r*_As_	0.41 (−1.00 to 1.00)			
*r*_Es_	0.15 (0.00 to 0.30)			
Generalized anxiety				
*r*_phs_	0.27 (0.16 to 0.37)	0.38 (0.29 to 0.47)		
*r*_As_	−0.13 (1.00 to 1.00)	0.85 (−1.00 to 1.00)		
*r*_Es_	0.29 (0.13 to 0.41)	0.38 (0.23 to 0.52)		
Separation anxiety				
*r*_phs_	−0.12 (−0.20 to 0.04)	0.02 (−0.06 to 0.09)	0.11 (0.02 to 0.20)	
*r*_As_	−0.33 (−1.00 to 1.00)	−0.16 (−0.60 to 0.20)	0.04 (−1.00 to 1.00)	
*r*_Es_	−0.08 (−0.22 to 0.05)	0.09 (−0.06 to 0.23)	0.13 (−0.02 to 0.27)	
Social anxiety				
*r*_phs_	0.12 (0.01 to 0.22)	0.05 (−0.06 to 0.14)	0.16 (0.03 to 0.27)	0.03 (−0.06 to 0.11)
*r*_As_	−0.45 (−1.00 to 1.00)	−0.94 (−1.00 to −0.12)	−0.76 (−1.00 to 1.00)	0.48 (−0.27 to 0.99)
*r*_Es_	0.17 (0.03 to 0.31)	0.20 (0.05 to 0.33)	0.20 (0.06 to 0.36)	−0.05 (−0.19 to 0.09)
Time-specific influences at 20				
Panic				
*r*_phs_	0.36 (0.30 to 0.42)			
*r*_As_	0.72 (0.05 to 1.00)			
*r*_Es_	0.30 (0.19 to 0.40)			
Generalized anxiety				
*r*_phs_	0.42 (0.37 to 0.47)	0.44 (0.39 to 0.49)		
*r*_As_	0.45 (−0.15 to 0.79)	0.48 (−0.07 to 0.98)		
*r*_Es_	0.42 (0.31 to 0.51)	0.44 (0.35 to 0.53)		
Separation anxiety				
*r*_phs_	0.38 (0.32 to 0.44)	0.44 (0.38 to 0.50)	0.47 (0.41 to 0.52)	
*r*_As_	0.66 (0.15 to 0.98)	0.61 (−0.58 to 1.00)	0.63 (−0.01 to 0.95)	
*r*_Es_	0.32 (0.21 to 0.42)	0.42 (0.32 to 0.52)	0.43 (0.33 to 0.53)	
Social anxiety				
*r*_phs_	0.45 (0.40 to 0.50)	0.47 (0.41 to 0.52)	0.56 (0.51 to 0.61)	0.43 (0.37 to 0.48)
*r*_As_	0.74 (0.31 to 0.99)	0.70 (−0.25 to 1.00)	0.69 (0.13 to 0.94)	0.33 (−0.43 to 0.79)
*r*_Es_	0.38 (0.28 to 0.48)	0.43 (0.34 to 0.53)	0.53 (0.45 to 0.62)	0.45 (0.34 to 0.55)

*r*_phl_, Phenotypic correlations between the latent factors; *r*_Al_, genetic correlations between the latent factors; *r*_El_, non-shared environmental correlations between the latent factors; *r*_phs_, phenotypic correlations between the time-specific influences; *r*_As_, genetic correlations between the time-specific influences; *r*_Es_, non-shared environmental correlations between the time-specific influences.

95% confidence intervals (CIs) are presented in parentheses. CIs not inclusive of zeros indicate significant correlations. Non-overlapping CIs mean significant difference between the values.

AE models are presented, as C influences were not significant and were dropped from the multivariate models without a significant deterioration of the fit (Supplementary Table S2). The AIC values suggest that dropping C lead to improvement of the model fit at these three waves. Full ACE results are presented in Supplementary Tables S3–S5 for completeness.

Model fit statistics for comparisons to the saturated models, and testing whether parameters can be dropped, are presented in Supplementary Table S2. Model fit statistics corroborate AE models and in the full models C estimates are very small. However, for completeness full ACE models are presented in Supplementary Tables S3–S5. Full ACE Cholesky decompositions suggest smaller genetic innovation than AE models (Supplementary Table S3). Otherwise dropping C from the models did not have impact on the interpretation of the results. The within-time analyses of these variables, including univariate ACE results, are presented elsewhere (Lau *et al.* 2007; Waszczuk *et al.* 2014). The longitudinal association between depression at times 1 and 2 was also reported previously (Lau & Eley, 2006).

## Discussion

The current study is the first to investigate how etiological influences contribute to developmental stability and change of depression, four anxiety symptom scales, and their co-occurrence across adolescence and young adulthood. The results provide support for largely stable and broad genetic influences accounting for co-occurrence and continuity over time. Environmental influences were generally more specific to time and symptom scales, contributing to change in symptoms over time.

### Homotypic continuity

#### Genetic influences on symptoms stability

Moderate homotypic continuity of depression and each anxiety symptom scale across the 5-year period was observed, as expected (Costello *et al.* 2003; Rutter *et al.* 2006; Gregory *et al.* 2007). We found that stable genetic influences largely underpinned this continuity, in agreement with previous research that suggests strong genetic stability across development (O'Connor *et al.* 1998; Gillespie *et al.* 2004; Trzaskowski *et al.* 2011; Garcia *et al.* 2013; Waszczuk *et al.* 2013).

#### Environmental and genetic influences on symptoms change

The non-shared environmental influences on homotypic continuity of each symptom were largely time-specific, as expected (Scourfield *et al.* 2003; Van Der Valk *et al.* 2003; Bartels *et al.* 2004; Haberstick *et al.* 2005; Lau & Eley, 2006; Zavos *et al.* 2012; Garcia *et al.* 2013; Lewis & Plomin, 2015). Furthermore, we found new genetic influences emerged over time (genetic innovation; Kendler *et al.* 2008*a*) and previous genetic influences gradually declined over time (genetic attenuation), in agreement with other findings (Scourfield *et al.* 2003; Van Der Valk *et al.* 2003; Bartels *et al.* 2004; Haberstick *et al.* 2005; Lau & Eley, 2006; Kendler *et al.* 2008*a*, *b*; Zavos *et al.* 2012; Nivard *et al.* 2015; Lewis & Plomin, 2015). These newly emerging, developmentally dynamic environmental and genetic effects can contribute to change in the course of depression and anxiety symptoms.

The current study extends previous findings by investigating longitudinal etiological influences on homotypic continuity of depression and anxiety symptoms scales separately. A similar pattern of substantial genetic stability and largely time-specific environmental influences was observed on all symptoms, possibly due to a substantial overlap between the genes influencing depression and anxiety (Kendler *et al.* 1987; Thapar & McGuffin, 1997; Eley & Stevenson, 1999; Mosing *et al.* 2009; Zavos *et al.* 2012; Waszczuk *et al.* 2014). However, some differences were notable. Depression, generalized and social anxiety symptoms showed more genetic stability than panic and separation anxiety symptoms, where genetic influences tended to attenuate more sharply, with proportionately greater genetic innovation at age 17 (panic and separation anxiety symptoms) and 20 years (separation anxiety symptoms). This might reflect relatively late median age of onset of panic disorder (Costello *et al.* 2003; Kessler *et al.* 2005*a*), and that pediatric and adult-onset separation anxiety might differ considerably (Shear *et al.* 2006; Costello *et al.* 2011).

### Heterotypic continuity

#### Genetic influences on symptoms stability

Heterotypic continuity across the symptom scales was significant, reflecting high co-morbidity between depression and anxiety symptoms (Merikangas, 1993; Pine *et al.* 2001; Costello *et al.* 2003; Goodwin *et al.* 2004; Rutter *et al.* 2006; Ferdinand *et al.* 2007; Gregory *et al.* 2007; Moffitt *et al.* 2007; Spatola *et al.* 2007; Trzaskowski *et al.* 2011). This longitudinal co-morbidity was largely explained by genetic overlap between the stable genetic influences that contribute to chronicity of each disorder, as well as overlap between the time-specific genetic influences. The time-specific influences represent developmentally dynamic genes that operate across short time periods and might reflect genes that come online in late adolescence or young adulthood. The current study provides preliminary evidence that both stable and time-specific genetic influences have general effects (i.e. on both depression and anxiety) (Eley, 1997), contributing to the enduring high genetic overlap between the symptom scales over time. These results carry implications for molecular genetic studies of depression and anxiety that aim to identify specific genetic variants involved in these disorders. They provide preliminary support for broadening the phenotypes included in molecular genetic studies, to increase power to detect shared susceptibility loci for a range of internalizing symptoms (O'Reilly *et al.* 2012; Hettema *et al.* 2015). However, the developmentally dynamic nature of genetic influences, in particular the genetic attenuation and innovation seen in adolescence suggests that stratifying sample collection by age may reduce heterogeneity (Zaitlen *et al.* 2012; Traylor *et al.* 2015). Identifying specific genes or polygenic risk scores may in turn inform clinical interventions, for example by using genetic markers to predict pharmacological or psychological treatment response (Keers & Aitchison, 2011; Eley *et al.* 2012; Lester & Eley, 2013).

#### Environmental influences on symptoms change and stability

As expected, environmental influences were largely time- and symptom-specific, thus contributing to the *change* in co-morbidity over time. However, a modest proportion of environmental influences contributed significantly to the stability of each symptom scale, albeit to a lesser extent than the genetic influences. The results are in line with some previous findings (O'Connor *et al.* 1998; Kendler *et al.* 2008*a*; Nivard *et al.* 2015), and extend them by showing that these stable non-shared environmental influences overlap considerably between depression and anxiety symptom scales, contributing to longitudinal co-morbidity. The results indicate that some environmental influences play a significant role in maintenance of depression and anxiety alongside genetic influences, possibly by producing enduring effects through biological and social changes in an individual (Kendler *et al.* 2011). These enduring environmental influences have an impact on a wide range of outcomes. These may include effects of severe environmental stressors such as childhood maltreatment or natural disasters (Kendler *et al.* 2000; Goenjian *et al.* 2005; Anda *et al.* 2006; Asselmann *et al.* 2015). Future studies should identify the life events that operate in this stable and broad manner to inform transdiagnostic interventions and prevention strategies (Barlow *et al.* 2004; Clark & Taylor, 2009; McEvoy *et al.* 2009; Wilamowska *et al.* 2010; Weersing *et al.* 2012; Krueger & Eaton, 2015).

### Limitations

The genetically informative, representative sample and multiple time points are strengths of the current study. However, a number of limitations are noteworthy. First, our analyses used only self-report symptom scales and the results should be replicated in clinical samples with co-morbid diagnoses and using lifetime diagnostic interviews. This approach was taken because clinical levels of internalizing disorders are rare in general adolescent population and questionnaires might capture less severe symptoms of these disorders, for example self-reported panic might capture physical symptoms of anxiety rather than panic attacks. However, symptoms of internalizing disorders are important markers of psychopathology (Pickles *et al.* 2001; Fergusson *et al.* 2005; Balázs *et al.* 2013). Common mental disorders are now considered to be the extremes of quantitative traits (Plomin *et al.* 2009; Insel *et al.* 2010) and there is evidence that differently defined internalizing problems have the same etiology (Kendler *et al.* 1987; Kendler *et al.* 1992*a*, *b*). Second, at time 3 a different anxiety questionnaire was used reflecting the participants’ older age. However, the longitudinal associations suggest a comparable continuity of the scores within and across different measures, in line with the view that they measure the same underlying constructs. Third, there was attrition in the sample. Although attrition bias might complicate estimation of trait prevalence, it is unlikely to affect the estimation of between trait associations (Wolke *et al.* 2009). Fourth, we did not measure other anxiety symptoms such as phobias, and future research should extend our findings to a wider range of internalizing symptoms. Fifth, future work should explore other types of continuity that were not addressed here, such as continuity across diagnoses. Last, there are limitations inherent to the twin design, discussed comprehensively elsewhere (Plomin *et al.* 2013). These have minimal and contrasting effects on parameter estimates which should be taken as indicative rather than absolute.

## Conclusions

Our results suggest that both homotypic and heterotypic continuity of depression and anxiety symptoms across adolescence and young adulthood is underpinned largely by stable genetic influences, while non-shared environmental effects tend to be time- and symptom-specific. The results have multiple implications for future molecular genetics research and clinical practice in the context of co-morbidity. They affirm the need to continue examining how the risk and maintenance factors for internalizing psychopathology operate across development to inform successful prevention and intervention strategies.

## Supplementary material

For supplementary material accompanying this paper visit http://dx.doi.org/10.1017/S0033291715001634. click here to view supplementary material
