# Supplementary material for: The stability and change of etiological influences on depression, anxiety symptoms and their co-occurrence across adolescence and young adulthood
Source: Psychol Med. 2015 Aug 27;46(1):161–75. doi: 10.1017/S0033291715001634 (PMC4673666; doi:10.1017/S0033291715001634)
Supplement: Supplementary file 1 [file S0033291715001634sup001.docx]

**Supplementary Methods – Recruitment details**

The G1219 study is a longitudinal study of 3,640 adolescent twins and siblings. The sample was recruited from two sources. First, adolescent offspring of adults from a large-scale population-based study (GENESIS, [Sham et al. (2000](#_ENREF_5))) were invited to participate in this or another study ([Curran et al., 2003](#_ENREF_1)). Of the 3,600 responses, 1,818 adolescents (51%) from 1,294 families agreed to participate in G1219. Second, a random selection of live twin births born between 1985 and 1988 identified by the UK Office of National Statistics were recruited by Heath Authorities and General Practitioners on behalf of G1219 team. Of the 2,947 families contacted, 1,381 (47%) participated. Only respondents aged 12 to 19 were included within the final sample. The present analyses focus on waves 2-4 of the data collection, when the participants were on average 15, 17 and 20 years old. Please note that we refer to these waves as Times 1-3 in our study for ease of presentation. Zygosity was established using parent-report questionnaires assessing the physical similarity between pairs. This method is estimated to be over 95% accurate ([Goldsmith, 1991](#_ENREF_2), [Price et al., 2000](#_ENREF_4)). When there was disagreement between zygosity ratings between wave one and two, DNA was obtained (N=26 pairs) before final classifications were made. The social-economic status (SES) of G1219 participants was somewhat higher than a population based sample, with 39% educated to A-level or above compared to 32% in the nationally representative sample ([Meltzer et al., 2000](#_ENREF_3)). Parents from the G1219 sample were also more likely to own their own homes (82% compared to 68%) than the nationally representative sample.

For all waves, informed consent was obtained from parents/guardians of all participating adolescents under 16 and from participants themselves when over 16. The study was granted ethical approval by the Research Ethics Committees of the Institute of Psychiatry, South London and Maudsley NHS Trust for all waves, and Goldsmiths, University of London at wave 4.

**Table S1 – Longitudinal Cholesky results with confidence intervals**

| **Path** | **Depression** | **Panic** | **Generalized**  **Anxiety** | **Separation**  **Anxiety** | **Social**  **Phobia** |
| --- | --- | --- | --- | --- | --- |
| a1_1_ | .52 (.46-.58) | .40 (.33-.46) | .45 (.38-.51) | .44 (.37-.50) | .42 (.35-.49) |
| a1_2_ | .29 (.21-.37) | .17 (.09-.26) | .21 (.13-.31) | .14 (.08-.22) | .23 (.15-.32) |
| a1_3_ | .26 (.17-.35) | .20 (.12-.30) | .18 (.11-.27) | .12 (.06-.20) | .27 (.18-.37) |
| a2_2_ | .18 (.09-.26) | .25 (.15-.35) | .18 (.08-.28) | .27 (.16-.36) | .15 (.07-.23) |
| a2_3_ | .01 (.00-.06) | .08 (.01-.17) | .10 (.02-.21) | .01 (.00-.06) | .08 (.01-.18) |
| a3_3_ | .13 (.03-.22) | .07 (.00-.16) | .11 (.01-.20) | .23 (.13-.32) | .09 (.00-.17) |
| e1_1_ | .48 (.42-.54) | .60 (.54-.67) | .55 (.49-.62) | .56 (.50-.63) | .58 (.51-.65) |
| e1_2_ | .02 (.01-.05) | .03 (.01-.07) | .05 (.02-.09) | .01 (.00-.03) | .08 (.04-.12) |
| e1_3_ | .00 (.00-.02) | .01 (.00-.03) | .00 (.00-.02) | .03 (.01-.06) | .02 (.00-.04) |
| e2_2_ | .51 (.44-.59) | .54 (.46-.64) | .56 (.47-.65) | .59 (.50-.68) | .54 (.47-.62) |
| e2_3_ | .06 (.03-.10) | .03 (.00-.07) | .05 (.02-.10) | .01 (.00-.03) | .06 (.03-.10) |
| e3_3_ | .55 (.47-.64) | .61 (.53-.70) | .56 (.48-.65) | .61 (.52-.71) | .49 (.42-.57) |

**Table S1 (continued) – Longitudinal Cholesky results with confidence intervals**

*Notes:*

*a1_1-3_* and *e1_1-3_* – proportion of total variance accounted for by the genetic/environmental factor (A1/E1) that emerged at time 1 (age 15) on the variables at each time point (ages 15, 17 and 20) (specific time point denoted by subscript).

*a2_2-3_* and *e2_2-3_* – proportion of total variance accounted for by the genetic/environmental factor (A2/E2) that emerged at time 2 (age 17) on the variables at time points 2 (age 17) and 3 (age 20) (specific time point denoted by subscript).

*a3_3_* and *e3_3_* – proportion of total variance accounted for by the genetic/environmental factor (A3/E3) that emerged at time 3 (age 20) on the variables at time 3 (age 20).

All paths are squared. Square root of these values should be taken to obtain variance paths.

95% Confidence Intervals (CIs) are presented in brackets. CIs not inclusive of zeros indicate significant correlations. Non-overlapping CIs mean significant difference between the values.

AE models are presented, as C influences were not significant and were dropped from the multivariate models without a significant deterioration of the fit (Table S2). The AIC values suggest that dropping C lead to improvement of the model fit at these three waves. Full ACE results are presented in Tables S3-5 for completeness.

Univariate results have been presented before ([Waszczuk et al., 2014](#_ENREF_7)), but can also be calculated by adding all the paths contributing to the variable (e.g. heritability of depression at time 3 is a1_3_+ a2_3_+ a3_3_=.40).

|  |  |  | **Comparison to Saturated Model** | | | **Comparison to Cholesky decomposition (ACE)** | | |  | |
| --- | --- | --- | --- | --- | --- | --- | --- | --- | --- | --- |
|  | **-2LL** | **df** | **χ^2^** | **Δ df** | **p-value** | **χ^2^** | **Δ df** | **p-value** | **AIC** | **BIC (size-adjusted)** |
| **Depression** | | | | | | | | | | |
| Saturated model | 9584.49 | 5408 |  |  |  |  |  |  | -1231.51 | 10655.14 |
| Cholesky decomposition (ACE) | 9923.09 | 5600 | 338.60 | 192 | <.05 |  |  |  | -1276.91 | 10042.05 |
| **Cholesky decomposition (AE)** | **9925.37** | **5606** | **340.88** | **198** | **<.05** | **2.28** | **6** | **.89** | **-1286.63** | **10014.59** |
| Cholesky decomposition (CE) | 9971.37 | 5606 | 386.88 | 198 | <.05 | 48.28 | 6 | <.05 | -1240.63 | 10060.59 |
| Cholesky decomposition (E) | 10196.61 | 5612 | 612.12 | 204 | <.05 | 273.52 | 12 | <.05 | -1027.39 | 10256.09 |
| **Panic** | | | | | | | | | | |
| Saturated model | 11382.69 | 5375 |  |  |  |  |  |  | 632.69 | 10897.22 |
| Cholesky decomposition (ACE) | 11653.70 | 5567 | 271.01 | 192 | <.05 |  |  |  | 519.70 | 11772.66 |
| **Cholesky decomposition (AE)** | **11656.51** | **5573** | **273.82** | **198** | **<.05** | **2.80** | **6** | **.83** | **510.51** | **11745.73** |
| Cholesky decomposition (CE) | 11687.32 | 5573 | 304.63 | 198 | <.05 | 33.62 | 6 | <.05 | 541.32 | 11776.54 |
| Cholesky decomposition (E) | 11833.37 | 5579 | 450.68 | 204 | <.05 | 179.67 | 12 | <.05 | 675.37 | 11892.85 |
| **Generalized Anxiety** | | | | | | | | | | |
| Saturated model | 26527.66 | 5379 |  |  |  |  |  |  | 15769.66 | 27598.31 |
| Cholesky decomposition (ACE) | 26785.26 | 5571 | 257.60 | 192 | <.05 |  |  |  | 15643.26 | 26904.22 |
| **Cholesky decomposition (AE)** | **26786.55** | **5577** | **285.89** | **198** | **<.05** | **1.29** | **6** | **.97** | **15632.55** | **26875.77** |
| Cholesky decomposition (CE) | 26818.16 | 5577 | 290.51 | 198 | <.05 | 32.90 | 6 | <.05 | 15664.16 | 26907.38 |
| Cholesky decomposition (E) | 26970.31 | 5583 | 442.65 | 204 | <.05 | 185.05 | 12 | <.05 | 15804.31 | 27029.79 |
| **Separation Anxiety** | | | | | | | | | | |
| Saturated model | 7599.95 | 5381 |  |  |  |  |  |  | -3162.05 | 8670.60 |
| Cholesky decomposition (ACE) | 7886.44 | 5573 | 286.49 | 192 | <.05 |  |  |  | -3259.56 | 8005.41 |
| **Cholesky decomposition (AE)** | **7890.10** | **5579** | **290.14** | **198** | **<.05** | **3.65** | **6** | **.72** | **-3267.91** | **7979.32** |
| Cholesky decomposition (CE) | 7923.74 | 5579 | 323.78 | 198 | <.05 | 37.30 | 6 | <.05 | -3234.26 | 8012.96 |
| Cholesky decomposition (E) | 8095.58 | 5585 | 495.63 | 204 | <.05 | 209.14 | 12 | <.05 | -3074.42 | 8155.06 |
| **Social Anxiety** | | | | | | | | | | |
| Saturated model | 28449.24 | 5387 |  |  |  |  |  |  | 17675.24 | 29519.89 |
| Cholesky decomposition (ACE) | 28693.82 | 5582 | 244.58 | 192 | <.05 |  |  |  | 17529.82 | 28797.91 |
| **Cholesky decomposition (AE)** | **28694.79** | **5588** | **245.55** | **198** | **<.05** | **0.96** | **6** | **.99** | **17518.79** | **28769.14** |
| Cholesky decomposition (CE) | 28727.05 | 5588 | 277.81 | 198 | <.05 | 33.22 | 6 | <.05 | 17551.05 | 28801.40 |
| Cholesky decomposition (E) | 28872.23 | 5594 | 422.99 | 204 | <.05 | 178.40 | 12 | <.05 | 17684.23 | 28916.84 |
| **All variables** | | | | | | | | | | |
| Saturated model | 73044.58 | 25535 |  |  |  |  |  |  | 21974.58 | 84758.52 |
| Common pathway model (ACE) | 76373.36 | 27793 | 3328.78 | 2258 | <.05 |  |  |  | 20787.36 | 77473.75 |
| **Common pathway model (AE)** | **76381.08** | **27853** | 3336.50 | 2318 | **<.05** | **7.72** | **60** | **1.00** | **20675.08** | **77184.07** |
| Common pathway model (CE) | 76498.78 | 27853 | 3454.20 | 2318 | <.05 | 125.42 | 60 | <.05 | 20792.78 | 77301.77 |
| Common pathway model (E) | 77138.64 | 27913 | 4094.06 | 2378 | <.05 | 765.28 | 120 | <.05 | 21312.64 | 77644.22 |

**Table S2 - Model fit statistics for longitudinal Cholesky decompositions and Common pathway model**

**Table S2 (continued) - Model fit statistics for longitudinal Cholesky decompositions and Common pathway model**

*Notes:*

*-2LL* – minus twice the log likelihood; *df*- degrees of freedom; *Δ df* – degrees of freedom difference; *p* – probability; *AIC* – Akaike’s information criterion; *BIC* – Bayesian’s information criterion.

The best fitting model (shown in bold) was selected based on the principle of parsimony and lowest AIC and BIC value. A difference in AIC between two models of 2 or less, provides equivalent support for both models (in which case the most parsimonious model should be chosen), a difference of 3 indicates that the lower AIC model has considerably more support and a difference of more than 10, indicates that the lower AIC model is a substantially better fit compared to the higher AIC model ([Wagenmakers and Farrell, 2004](#_ENREF_6)). Shared-environmental, but not genetic influences can be dropped from the models without significant deterioration of the fit. The AIC and BIC values suggest that dropping C lead to improvement of the model fit at these three waves.

The Cholesky decompositions and common pathway model were significantly different from the corresponding saturated models, indicating poor fit, however this is common in studies with large sample sizes because minimal variance differences between groups can be highly statistically significant.

Quantitative sex differences imply that genetic and environmental influences differ in magnitude across sex whilst scalar sex differences indicate variance differences between males and females. Scalar sex differences were evident for all variables apart from social concerns at times 1-3, suggesting that males and females showed different variance on most measures. To account for these differences, a scalar was fitted in all twin modeling analyses at these time points.

**Table S3 – Longitudinal Cholesky results with confidence intervals (full ACE model)**

| **Path** | **Depression** | **Panic** | **Generalized**  **Anxiety** | **Separation**  **Anxiety** | **Social**  **Phobia** |
| --- | --- | --- | --- | --- | --- |
| a1_1_ | .45 (.29-.56) | .30 (.13-.44) | .41 (.23-.51) | .28 (.12-.45) | .42 (.29-.49) |
| a1_2_ | .38 (.19-.50) | .17 (.05-.38) | .21 (.08-.38) | .22 (.05-.44) | .23 (.09-.37) |
| a1_3_ | .34 (.18-.45) | .31 (.11-.43) | .26 (.07-.43) | .26 (.06-.42) | .27 (.13-.42) |
| a2_2_ | .08 (.00-.24) | .24 (.05-.35) | .18 (.02-.27) | .19 (.00-.42) | .11 (.00-.22) |
| a2_3_ | .05 (.00-.19) | .04 (.01-.16) | .06 (.00-.24) | .01 (.00-.03) | .16 (.00-.26) |
| a3_3_ | .00 (.00-.20) | .00 (.00-.13) | .00 (.00-.18) | .07 (.00-.29) | .00 (.00-.16) |
| c1_1_ | .06 (.00-.18) | .08 (.00-.21) | .03 (.00-.16) | .12 (.00-.24) | .00 (.00-.09) |
| c1_2_ | .01 (.00-.14) | .01 (.00-.09) | .00 (.00-.10) | .00 (.00-.06) | .04 (.00-.18) |
| c1_3_ | .00 (.00-.09) | .00 (.00-.10) | .04 (.00-.18) | .01 (.00-.09) | .01 (.00-.16) |
| c2_2_ | .00 (.00-.14) | .00 (.37-.05) | .00 (.00-.06) | .00 (.00-.09) | .00 (.00-.13) |
| c2_3_ | .00 (.00-.09) | .00 (.00-.11) | .00 (.00-.17) | .00 (.00-.11) | .00 (.00-.13) |
| c3_3_ | .00 (.00-.07) | .00 (.00-.10) | .00 (.00-.13) | .00 (.00-.11) | .00 (.00-.11) |
| e1_1_ | .49 (.43-.56) | .62 (.55-.70) | .56 (.49-.64) | .60 (.52-.67) | .58 (.51-.65) |
| e1_2_ | .02 (.01-.05) | .04 (.01-.07) | .05 (.02-.09) | .01 (.00-.03) | .07 (.04-.12) |
| e1_3_ | .00 (.00-.01) | .01 (.00-.03) | .00 (.00-.02) | .02 (.01-.06) | .02 (.00-.04) |
| e2_2_ | .52 (.44-.59) | .54 (.46-.64) | .56 (.47-.65) | .59 (.50-.68) | .55 (.47-.63) |
| e2_3_ | .06 (.02-.11) | .03 (.00-.07) | .05 (.02-.10) | .01 (.00-.03) | .05 (.03-.10) |
| e3_3_ | .55 (.47-.63) | .61 (.53-.69) | .58 (.49-.66) | .62 (.53-.72) | .50 (.42-.57) |

**Table S3 (Continued) – Longitudinal Cholesky results with confidence intervals (full ACE model)**

*Notes:*

*a1_1-3_ , c1_1-3_* and *e1_1-3_* – proportion of total variance accounted for by the genetic/shared environmental/ non-shared environmental factor (A1/C1/E1) that emerged at time 1 (age 15) on the variables at each time point (ages 15, 17 and 20) (specific time point denoted by subscript).

*a2_2-3_* , *c2_2-3_* and *e2_2-3_* – proportion of total variance accounted for by the genetic/shared environmental/ non-shared environmental factor (A2/C2/E2) that emerged at time 2 (age 17) on the variables at time points 2 (age 17) and 3 (age 20) (specific time point denoted by subscript).

*a3_3_* , *c3_3_* and *e3_3_* – proportion of total variance accounted for by the genetic/shared environmental/ non-shared environmental factor (A3/C3/E3) that emerged at time 3 (age 20) on the variables at time 3 (age 20).

All paths are squared. Square root of these values should be taken to obtain variance paths.

95% Confidence Intervals (CIs) are presented in brackets. CIs not inclusive of zeros indicate significant correlations. Non-overlapping CIs mean significant difference between the values.

Univariate results have been presented before ([Waszczuk et al., 2014](#_ENREF_7)), but can also be calculated by adding all the paths contributing to the variable (e.g. heritability of depression at time 3 is a1_3_+ a2_3_+ a3_3_=.39).

**Table S4**– Common pathway model results (full ACE model): Genetic and environmental influences on the latent factor, and latent factor and time-specific influences on each variable.

|  |  | Depression | | | Panic | | | Generalized Anxiety | | | Separation Anxiety | | | Social Anxiety | | |
| --- | --- | --- | --- | --- | --- | --- | --- | --- | --- | --- | --- | --- | --- | --- | --- | --- |
| Etiological influences on the latent factor | A_l_ | .76  (.54-.84) | | | .66  (.42-.77) | | | .63  (.36-.74) | | | .69  (.40-.83) | | | .60  (.33-.71) | | |
|  | C_l_ | .00  (.00-.16) | | | .00  (.00-.17) | | | .00  (.00-.19) | | | .01  (.00-.21) | | | .01  (.00-.21) | | |
|  | E_l_ | .24  (.16-.35) | | | .34  (.23-.46) | | | .37  (.26-.49) | | | .31  (.17-.46) | | | .39  (.29-.51) | | |
| Mean age |  | 15 | 17 | 20 | 15 | 17 | 20 | 15 | 17 | 20 | 15 | 17 | 20 | 15 | 17 | 20 |
| Latent factor influences on each variable | L | .63  (.59-.67) | .78  (.73-.82) | .58  (.53-.62) | .56  (.52-.60) | .71  (.66-.76) | .62  (.57-.66) | .57  (.53-.61) | .82  (.78-86) | .58  (.53-.63) | .55  (.51-.60) | .46  (.39-.52) | .60  (.55-.66) | .62  (.58-.65) | .82  (.78-.86) | .68  (.64-.72) |
| Time-specific etiological influences on each variable | A_s_ | .11  (.00-.25) | .02  (.00-.10) | .13  (.00-.22) | .10  (.00-.22) | .11  (.02-.20) | .05  (.00-.14) | .21  (.05-.29) | .02  (.00-.08) | .07  (.00-.19) | .10  (.00-.25) | .24  (.08-.35) | .10  (.00-.20) | .15  (.04-.23) | .02  (.00-.06) | .09  (.00-.17) |
|  | C_s_ | .07  (.00-.16) | .02  (.00-.07) | .00  (.00-.10) | .06  (.00-.15) | .01  (.00-.06) | .02  (.00-.10) | .01  (.00-.12) | .00  (.00-.03) | .04  (.00-.14) | .08  (.00-.18) | .01  (.00-.11) | .00  (.00-.11) | .01  (.00-.09) | .00  (.00-.04) | .00  (.00-.11) |
|  | E_s_ | .43  (.36-.49) | .35  (.28-.42) | .53  (.45-.63) | .53  (.46-.60) | .38  (.29-.46) | .56  (.47-.64) | .46  (.39-.53) | .31  (.23-.38) | .55  (.46-.64) | .51  (.43-.58) | .54  (.45-.64) | .53  (.44-.63) | .46  (.39-.53) | .30  (.24-.37) | .44  (.37-.53) |

*Notes:*

A - additive genetic effects; C – shared environmental effects; E - non-shared environmental effects; L – Latent factor.

Mean ages provided in the headings.

**Table S4 (continued)**– Common pathway model results (full ACE model): Genetic and environmental influences on the latent factor, and latent factor and time-specific influences on each variable.

95% Confidence Intervals (CIs) are presented in brackets. CIs not inclusive of zeros indicate significant influences. Non-overlapping CIs mean significant difference between the values.

L needs to be squared to inform about the proportion of total variance accounted for by the latent factor. L^2^ should be multiplied by A_l_/ C_l_ /E_l_ to obtain the proportion of the total variance due to the genetic/shared environmental/non-shared environmental influences from the latent factor, respectively. Total variance of a trait = L^2^ + A_s_ + C_s_ +E_s_

**Table S5**– Common pathway model results (full ACE model): Phenotypic, genetic and environmental correlations between the latent factors and time-specific influences at 15, 17 and 20 years

|  | | Depression | Panic | Generalized Anxiety | Separation Anxiety |
| --- | --- | --- | --- | --- | --- |
| Latent Factors | | | | | |
| Panic | r_phl_ | .72 (.66-.80) |  |  |  |
|  | r_Al_ | .75 (.64-.92) |  |  |  |
|  | r_Cl_ | 1.00 (-.58-1.00) |  |  |  |
|  | r_El_ | .66 (.44-.85) |  |  |  |
| Generalized Anxiety | r_phl_ | .74 (.68-.80) | .83 (.78-.89) |  |  |
|  | r_Al_ | .81 (.70-.94) | .87 (.77-.99) |  |  |
|  | r_Cl_ | 1.00 (-1.00-1.00) | 1.00 (-1.00-1.00) |  |  |
|  | r_El_ | .60 (.41-.76) | .75 (.60-.88) |  |  |
| Separation Anxiety | r_phl_ | .58 (.50-.69) | .76 (.69-.82) | .80 (.74-.88) |  |
|  | r_Al_ | .60 (.44-.81) | .78 (.63-.90) | .86 (.74-1.00) |  |
|  | r_Cl_ | 1.00 (-.89-1.00) | 1.00 (-1.00-1.00) | 1.00 (-1.00-1.00) |  |
|  | r_El_ | .55 (.27-.80) | .73 (.50-.95) | .69 (.49-.88) |  |
| Social Anxiety | r_phl_ | .63 (.56-.71) | .62 (.54-.69) | .75 (.69-.81) | .64 (.56-.71) |
|  | r_Al_ | .68 (.54-.86) | .68 (.49-.83) | .77 (.62-.91) | .75 (.58-.92) |
|  | r_Cl_ | 1.00 (-1.00-1.00) | 1.00 (-1.00-1.00) | 1.00 (-1.00-1.00) | 1.00 (-1.00-1.00) |
|  | r_El_ | .56 (.37-.73) | .52 (.34-.68) | .71 (.57-.83) | .46 (.24-.66) |
| Time-specific influences at 15 | | | | | |
| Panic | r_phs_ | .41 (.29-.49) |  |  |  |
|  | r_As_ | .96 (-1.00-1.00) |  |  |  |
|  | r_Cs_ | .58 (68-1.00) |  |  |  |
|  | r_Es_ | .30 (.21-.39) |  |  |  |
| Generalized Anxiety | r_phs_ | .44 (.33-.52) | .47 (.38-.54) |  |  |
|  | r_As_ | .64 (-.75-1.00) | .52 (-1.00-1.00) |  |  |
|  | r_Cs_ | .58 (-1.00-1.00) | 1.00(-1.00-1.00) |  |  |
|  | r_Es_ | .38 (.28-.46) | .47 (.38-.54) |  |  |
| Separation Anxiety | r_phs_ | .31 (.19-.43) | .34 (.24-.44) | .41 (.31-.51) |  |
|  | r_As_ | .22 (-1.00-1.00) | .30 (-1.00-1.00) | .58 (-1.00-1.00) |  |
|  | r_Cs_ | .57 (-1.00-1.00) | 1.00(-1.00-1.00) | 1.00(-1.00-1.00) |  |
|  | r_Es_ | .33 (.23-.42) | .28 (.19-.37) | .37 (.27-.46) |  |
| Social Anxiety | r_phs_ | .34 (.24-.42) | .35 (.25-.42) | .48 (.40-.53) | .45 (.36-.53) |
|  | r_As_ | .59 (-.94-1.00) | .63 (-1.00-1.00) | .80 (.41-.99) | .69 (-1.00-1.00) |
|  | r_Cs_ | .88 (-1.00-1.00) | .90 (-1.00-1.00) | .90 (-1.00-1.00) | .90 (-1.00-1.00) |
|  | r_Es_ | .27 (.17-.37) | .28 (.18-.37) | .35 (.26-.44) | .39 (.30-.48) |
| Time-specific influences at 17 | | | | | |
| Panic | r_phs_ | .22 (.10-.32) |  |  |  |
|  | r_As_ | .82 (.42-1.00) |  |  |  |
|  | r_Cs_ | -1.00(-1.00-1.00) |  |  |  |
|  | r_Es_ | .15 (.01-.28) |  |  |  |
| Generalized Anxiety | r_phs_ | .28 (.16-.39) | .39 (.29-.49) |  |  |
|  | r_As_ | .46 (-1.00-1.00) | .42 (-1.00-1.00) |  |  |
|  | r_Cs_ | 1.00 (-1.00-1.00) | -.99(-1.00-1.00) |  |  |
|  | r_Es_ | .27 (.13-.40) | .40 (.25-.52) |  |  |
| Separation Anxiety | r_phs_ | -.09 (-.20--.01) | .00 (-.10-.09) | .11 (.01-.21) |  |
|  | r_As_ | -.15 (-1.00-.97) | -.26 (-1.00-.16) | .03 (-1.00-1.00) |  |
|  | r_Cs_ | -1.00(-1.00-1.00) | 1.00(-1.00-1.00) | -1.00(-1.00-1.00) |  |
|  | r_Es_ | -.09 (-.22-.04) | .10 (-.04-.23) | .14 (.00-.27) |  |
| Social Anxiety | r_phs_ | .10 (-.01-.22) | .06 (-.06-.17) | .16 (.04-.28) | .05 (-.05-.14) |
|  | r_As_ | -.80 (-1.00-1.00) | -.84 (-1.00-.11) | -.64 (-1.00-1.00) | .56 (-.25-1.00) |
|  | r_Cs_ | 1.00 (-1.00-1.00) | -.99(-1.00-1.00) | 1.00(-1.00-1.00) | -1.00 (-1.00-1.00) |
|  | r_Es_ | .17 (.03-.29) | .20 (.06-.32) | .22 (.06-.35) | -.05 (-.18-.08) |
| Time-specific influences at 20 | | | | | |
| Panic | r_phs_ | .36 (.26-.43) |  |  |  |
|  | r_As_ | .74 (-1.00-1.00) |  |  |  |
|  | r_Cs_ | .88 (.66-1.00) |  |  |  |
|  | r_Es_ | .31 (.20-.41) |  |  |  |
| Generalized Anxiety | r_phs_ | .44 (.33-.51) | .48 (.37-.54) |  |  |
|  | r_As_ | .63 (-1.00-1.00) | .92 (-1.00-1.00) |  |  |
|  | r_Cs_ | -.20 (-1.00-1.00) | -.44 (-1.00-1.00) |  |  |
|  | r_Es_ | .41 (.31-.50) | .44 (.35-.52) |  |  |
| Separation Anxiety | r_phs_ | .39 (.29-.46) | .46 (.36-.53) | .46 (.36-.54) |  |
|  | r_As_ | .71 (-.91-1.00) | .87 (-1.00-1.00) | .60 (-1.00-1.00) |  |
|  | r_Cs_ | -30 (-1.00-1.00) | -.48 (-1.00-1.00) | .99 (-1.00-1.00) |  |
|  | r_Es_ | .32 (.21-.42) | .41 (.32-.51) | .44 (.35-.53) |  |
| Social Anxiety | r_phs_ | .45 (.40-.50) | .46 (.37-.54) | .58 (.48-.64) | .43 (.33-.53) |
|  | r_As_ | .73 (-1.00-1.00) | .74 (-1.00-1.00) | .90 (-1.00-1.00) | .37 (-1.00-1.00) |
|  | r_Cs_ | .99 (-.12-1.00) | .83 (-1.00-1.00) | -.14 (-1.00-1.00) | -.24 (-1.00-1.00) |
|  | r_Es_ | .39 (.28-.48) | .43 (.34-.53) | .53 (.45-.62) | .45 (.34-.55) |

*Notes:*

r_phl_ - Phenotypic correlations between the latent factors; r_Al_ - Genetic correlations between the latent factors; r_Cl_ – Shared environmental correlations between the latent factors; r_El_ – Non-shared environmental correlations between the latent factors; r_phs_ - Phenotypic correlations between the time-specific influences; r_As_ - Genetic correlations between the time-specific influences; r_Cs_ – Shared environmental correlations between the time-specific influences; r_Es_ – Non-shared environmental correlations between the time-specific influences.

95% Confidence Intervals (CIs) are presented in brackets. CIs not inclusive of zeros indicate significant correlations. Non-overlapping CIs mean significant difference between the values.

**References**

**Curran, S., Mill, J., Sham, P., Rijsdijk, F., Marusic, K., Taylor, E. & Asherson, P.** 2003. CHIP: Defining a dimension of the vulnerability to Attention Deficit Hyperactivity Disorder (ADHD) using sibling and individual data of children in a community-based sample. *Behavior Genetics,* 119**,** 86-97.

**Goldsmith, H. H.** 1991. A zygosity questionnaire for young twins: A research note. *Behavior Genetics,* 21**,** 257-269.

**Meltzer, H., Gatward, R., Goodman, R. & Ford, T.** 2000. *Mental health of children and adolescents in Great Britain,* London, The Stationary Office.

**Price, T. S., Freeman, B., Craig, I. W., Petrill, S. A., Ebersole, L. & Plomin, R.** 2000. Infant zygosity can be assigned by parental report questionnaire data. *Twin Research,* 3**,** 129-133.

**Sham, P. C., Sterne, A., Purcell, S., Cherny, S. S., Webster, M., Rijsdijk, F. V., Asherson, P. J., Ball, D., Craig, I., Eley, T. C., Goldberg, D., Gray, J., Mann, A., Owen, M. & Plomin, R.** 2000. GENESiS: Creating a composite index of the vulnerability to anxiety and depression in a community-based sample of siblings. *Twin Research,* 3**,** 316-322.

**Wagenmakers, E.-J. & Farrell, S.** 2004. AIC model selection using Akaike weights. *Psychonomic bulletin & review,* 11**,** 192-196.

**Waszczuk, M. A., Zavos, H. M. S., Gregory, A. M. & Eley, T. C.** 2014. The phenotypic and etiological structure of depression and anxiety disorder symptoms in childhood, adolescence and young adulthood. *JAMA Psychiatry,* 71**,** 905-916.
